# Association Between Generalized Anxiety Disorder (GAD) and Polycystic Ovary Syndrome (PCOS) Diagnosed University‐Going Female Students in Bangladesh: A Cross‐Sectional Study

**DOI:** 10.1002/hsr2.72193

**Published:** 2026-03-22

**Authors:** Fardin Al Fahad Shakib, Ifratul Hasan, Mahmud Hossain, Shaherin Islam Tuba, Nowmy Niaz, Shamim Ahmed, Mohammad Borhan Uddin

**Affiliations:** ^1^ Department of Pharmaceutical Sciences, School of Health and Life Sciences North South University Dhaka Bangladesh; ^2^ Department of Statistics Shahjalal University of Science and Technology Sylhet Bangladesh

**Keywords:** Bangladesh, Bangladeshi female students, generalized anxiety disorder, mental health, polycystic ovary syndrome, risk factors

## Abstract

**Background and Aims:**

Generalized anxiety disorder (GAD) is a prevalent psychological burden among females of reproductive age diagnosed with polycystic ovary syndrome (PCOS). Bangladeshi university‐going PCOS‐diagnosed female students encounter unique stressors that may elevate anxiety symptoms when diagnosed with PCOS. Despite the known association between PCOS and anxiety, there is limited research on the sociodemographic and lifestyle risk factors of GAD in this population. This study examined the key risk factors associated with GAD among PCOS‐diagnosed students in Bangladesh.

**Methods:**

A cross‐sectional study was conducted from January to June 2024 across 15 public and private universities in Dhaka. A total of 282 female students aged 18–45 years, previously diagnosed with PCOS by a physician, were recruited using non‐probability sampling. Data were collected using a structured, expert‐validated questionnaire that included demographics, lifestyle, and psychological variables. The generalized anxiety disorder‐7 (GAD‐7) scale was used to assess anxiety. Associations were analyzed using Chi‐squared tests, and binary logistic regression was performed to identify significant predictors of GAD (*p*‐value < 0.05).

**Results:**

Of the 282 students, logistic regression analysis revealed that mood instability (AOR = 4.18, 95% CI: [1.78–10.6]), having children (AOR = 0.11, 95% CI: [0.01–0.76]), oral contraceptive pills (OCPs) (AOR = 1.99, 95% CI: [1.14–3.48]), skipping meals (AOR = 2.13, 95% CI: [1.18–3.90]), and age (AOR = 1.13, 95% CI: [1.06–1.22]) were significantly associated with GAD.

**Conclusion:**

The findings of this study highlight key health‐related factors, along with lifestyle, psychological, and risk factors that could potentially develop GAD in university students with PCOS. These study outcomes underscore the need for mental health and reproductive support programs in university settings, particularly in resource‐limited environments like Bangladesh.

## 1 Introduction

Polycystic ovary syndrome (PCOS) is one of the most common complex heterogeneous endocrine disorders in women of reproductive age [1]. This condition is diagnosed based on three primary criteria: ovulatory dysfunction, emergence of polycystic morphology of the ovaries, and increased androgen production [2]. Under the Rotterdam 2003 criteria and the NIH 1990 guidance, the prevalence of PCOS is 4%–21% globally, with 5.6% of cases involving Chinese women aged 19–45 [3]. Moreover, PCOS manifests diversely as prevalence rates spanning from 6% to 10% in the Western world to as high as 22.5% in the Indian subcontinent [4]. It has an exceptionally high burden in South Asian countries such as Bangladesh, where lifestyle changes and sociocultural factors contribute to its rising prevalence [5, 6]. Alongside the physiological (endocrine dysfunctions) [1]manifestations of PCOS, such as irregular menstruation, hirsutism, obesity, and insulin resistance, PCOS has profound psychological implications, including elevated risks of mood disorders, depression, and anxiety, as well as social status [5, 7, 8, 9].

The psychological burden of PCOS has garnered increasing attention from researchers in recent years [10, 11, 12]. Women with PCOS have a significantly higher prevalence of experiencing mental health disorders, including depression, anxiety, particularly generalized anxiety disorder (GAD), compared to their healthier counterparts [13, 14]. GAD is often characterized by chronic, excessive worry and physical symptoms such as restlessness and difficulty concentrating, which may severely impact academic performance, interpersonal relationships, and overall quality of life in young women [15, 16]. Social barriers, such as gender‐based discrimination, cultural expectations, and a lack of mental health assistance, may raise the risk of developing GAD with PCOS in South Asian nations like Bangladesh. More importantly, for university‐going female students from both undergraduate and postgraduate programs, who are already going through the academic stressors and transitional life phases, psychological distress can be exacerbated by the presence of PCOS. A meta‐analysis conducted by Veltman‐Verhulst et al. [17] found that women with PCOS have nearly a threefold increased risk of depression and a twofold higher risk of anxiety, thus underscoring the substantial mental health burden associated with this condition [18].

In the South Asian context, including Bangladesh, the intersection of PCOS and mental health is particularly concerning. This is because of socio‐cultural stigma, limited mental health services, and lack of awareness about women's reproductive health [5, 19]. While several studies documented the high prevalence rates of anxiety and depression among PCOS patients in Thailand [20]and the Philippines [21], there remains a significant gap in understanding the specific lifestyle and socio‐demographic risk factors of GAD in the Bangladeshi population.

It is well established that hormonal and metabolic disturbances are deeply connected with mood swings among women with PCOS. Women with PCOS also experience fertility troubles, and infertility is associated with emotional distress and anxiety disorders [22]. The physical manifestations of PCOS, such as hirsutism and obesity, are considered driving factors for anxiety and body image dissatisfaction [23].

In light of the increasing reports on anxiety and mental health concerns, there is a growing interest in understanding the association between GAD and PCOS, including identifying the specific risk factors that precipitate GAD in individuals diagnosed with PCOS. A cross‐sectional study found a link between PCOS patients' lower quality of life and the presence of depression. Additionally, it was discovered that PCOS patients had a higher prevalence of depressive and anxiety symptoms and a significantly poorer overall quality of life [24, 25]. Another study conducted in India showed that out of 192 north‐Indian women with PCOS, 25% had significant anxiety according to the GAD‐7 scale [26], a brief, self‐assessment scale that was initially developed to quantify GAD and has been validated for use in primary care settings within the United States [27].

The main aim of this study is to find significant risk factors that may be responsible for developing GAD in PCOS‐diagnosed female students in Bangladesh. These risk factors were investigated under the lifestyle and health characteristics of PCOS‐diagnosed women. Factors such as mood instability, contraceptive use, marital status, skipping meals, and family history of diabetes/high blood pressure (HBP) may influence psychological outcomes. However, these associations remain unexplored in the context of university students with PCOS in Bangladesh. A previous study by Hasan et al [5] showcased the associated risk factors for mental health problems among students, professionals, and business/self‐employed individuals with PCOS across Bangladesh. To the best of our knowledge from the literature review to date, our research is the first comprehensive study regarding GAD risk factors among PCOS‐diagnosed female students from different universities in Bangladesh through a cross‐sectional study. By focusing on young as well as middle‐aged, academically active populations, the study potentially seeks to provide insights into how reproductive health conditions like PCOS intersect with mental health challenges in educational settings. In addition, the outcomes from this research may inform targeted interventions and health policies that could essentially address the psychological needs of women with PCOS in Bangladesh. Furthermore, the data of this study may hold relevance for other South Asian countries as the lifestyle and health‐related factors of Bangladeshi women are similar to those of India, Pakistan, and Sri Lanka.

## 2 Methods

### 2.1 Study Design, Participants, and Setting

This cross‐sectional study design was employed to collect data through face‐to‐face, interviewer‐administered interviews from undergraduate and postgraduate university‐going female students at 15 different universities in Dhaka, Bangladesh. Two hundred eighty‐two participants who were diagnosed with PCOS in the age group of 18–45 years were included in the study. The setting for this study included eight government and seven private universities in Dhaka, the capital of Bangladesh, and was conducted from January 2024 to June 2024. This study adhered to the STROBE (Strengthening the Reporting of Observational Studies in Epidemiology) guidelines, which are appropriate for cross‐sectional studies, to ensure comprehensive and transparent reporting [28].

### 2.2 Data Collection and Questionnaire Design

Data were collected from eligible participants who had a physician‐confirmed diagnosis of PCOS, as documented in their prescriptions, which indicated diagnosis based on ultrasound reports. The study questionnaire was developed following an extensive review of previously validated questionnaires that investigated factors attributed to mental health problems, like anxiety and depression, among PCOS‐affected women [5, 25, 29]. At first, the questionnaire was prepared in English. We sought assistance from a pharmacy graduate, a medical graduate, and a non‐medical individual with expertise in English. Then, we conducted a pilot study to assess the reliability, involving 15 randomly selected female university students who were not included in the main study, to confirm the clarity and understanding of the questions. Finally, the questionnaire underwent expert review for content validity by subject matter experts. The consistency was assessed using Cronbach's alpha [30], which yielded a coefficient of 0.70, indicating an acceptable level of reliability that the questionnaire could be used to proceed with the main study. The final structured questionnaire (in English) was organized into five sections: demographics (age, education level, BMI, marital status), health status and diseases (conception status, family history of PCOS, diabetes/HBP), lifestyle‐related factors (physical exercise, junk food consumption, smoking habit), psychological factors (mood swings, loneliness), and risk factors, such as children status, oral contraceptive pills (OCPs) associated with GAD.

### 2.3 Study Variables

#### 2.3.1 Outcome Variable

The outcome variable in this study was the self‐reported “Status of GAD.” It is a binary variable, categorized as “yes” (coded as “1”) if a participant reported having a GAD 7‐item (GAD‐7) score of more than 10, and classified as “no” (coded as “0”) if they had a GAD‐7 score less than 10. The total GAD‐7 score ranges from 0 to 27, separated into four different categories indicating distinct level of anxiety: (0–4 – minimal anxiety, 5–9 – mild anxiety, 10–14 – moderate anxiety, and 15–21 – severe anxiety). The GAD‐7 scale is an efficient tool for evaluating generalized anxiety symptoms [15].

#### 2.3.2 Explanatory Variables

The study included a range of independent variables analyzed to assess their associations with the participants' GAD status. They were‐ age, education level, BMI category, religion, marital status, children status, conception status, miscarriage status, smoking habit, family history of PCOS, menstrual cycle, irregular periods, weight gain, mood swings, loneliness, lower back pain, weakness, family history of diabetes/HBP, physical exercise, junk food consumption, diet pattern, meals on time, skip meals, water drinking, birth Control, OCPs.

### 2.4 Ethical Approval and Informed Consent

This research study was conducted in conformity with the ethical principles of the Declaration of Helsinki. The study has been granted ethical approval by the North South University Institutional Review Board/Ethics Review Committee (Project Code: 2024/OR‐NSU/IRB/0903). Although data were collected from students enrolled at 15 different universities in Dhaka, the study did not involve recruitment through hospitals or use of institutional clinical infrastructure. Participants were approached directly in university settings. Therefore, additional institutional ethical approvals were not required. Before data collection, all participants provided written informed consent, ensuring their complete comprehension of the study's objectives, importance, and confidentiality protection. Although participants were asked to provide names and contact information, data were stored in password‐protected files accessible only to the research team.

### 2.5 Sample Size Estimation and Data Collection

**Figure 1:**
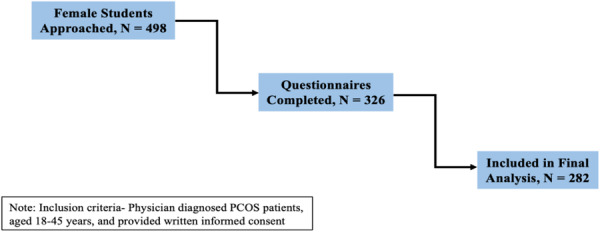
Participants recruitment flowchart.

To conduct our study, we used G*Power version 3.2 to calculate the sample size. Based on a statistical power of 80% and an alpha error probability of 5%, the minimum sample size required for this study was calculated to be 242 participants. We initially approached 498 female students, of whom 326 completed the questionnaire. Ultimately, this study enrolled a sufficient sample size of 282 students who satisfied the inclusion criteria and provided informed consent. Figure 1 presents the participant recruitment flowchart illustrating the screening and enrollment process. A non‐probability sampling approach was chosen to ensure the inclusion of cases relevant to the research objectives, allowing for focused investigation within a clinically defined subgroup.

### 2.6 Eligibility Criteria

#### 2.6.1 Inclusion Criteria

Female students of reproductive age (18–45 years) who have been diagnosed with PCOS by a physician were included in this study.

#### 2.6.2 Exclusion Criteria

Female students whose age was below 18 years or above 45 years, and who weren't diagnosed with PCOS by the physician, were excluded from the study. Additionally, individuals with a prior diagnosis of depression or those who were pregnant were omitted from participation in this research. The information was provided voluntarily by all participants.

### 2.7 Statistical Analysis

At first, exploratory data analysis was conducted to assess data distribution, detect missing values, and identify potential outliers. Descriptive statistics, including means and standard deviations for continuous variables and frequencies and percentages for categorical variables, were used to summarize the socio‐demographic, lifestyle‐related characteristics of the respondents. Graphical representations were also used to visualize key variables.

Bivariate analyses were performed to examine the association between the outcome variable GAD status: yes/no) and each independent variable. Pearson's chi‐squared tests were used for categorical predictors. Variables identified as potentially associated with GAD in the bivariate analysis were subsequently included in a multiple logistic regression model to determine factors independently associated with the likelihood of GAD among PCOS‐diagnosed university students. The logistic regression model was employed due to its ability to handle binary outcome variables.

A backward stepwise selection procedure based on the likelihood ratio test was applied, beginning with all 29 candidate predictors. Variables that did not meet the pre‐specified significance criterion were sequentially removed to obtain the final parsimonious model. Adjusted odds ratios (AORs) with 95% confidence intervals (CIs) and corresponding *p*‐values were reported to quantify the strength and precision of associations. Two‐sided tests were used throughout, with statistical significance set at *α* = 0.05.

All analyses were performed using R software version 4.4.2 (R Foundation for Statistical Computing, Vienna, Austria). The methodological approach followed the SAMPL (Statistical Analyses and Methods in the Published Literature) guidelines for transparent reporting of statistical results [31]. The statistical analysis and reporting in this manuscript followed the recommendations of Assel et al. (2019) on good statistical practices for clinical research reporting [32].

## 3 Results

### 3.1 Prevalence of Generalized Anxiety Disorder (GAD) Among the Participants

**Figure 2:**
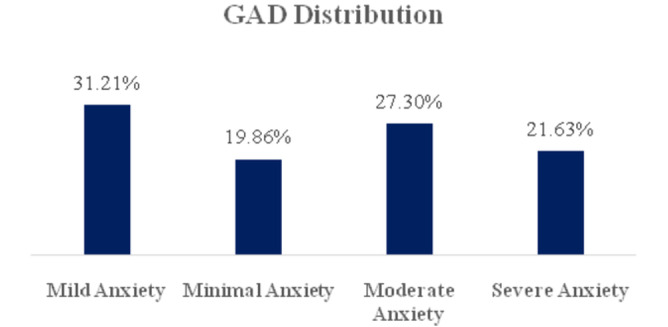
Prevalence and distribution of generalized anxiety disorder (GAD) among participants (*n* = 282).

Figure 2 illustrates the distribution of GAD levels among the participants. The majority (31.21%) experienced mild anxiety, followed by moderate anxiety (27.30%). Severe anxiety was reported by 21.63% of participants, while 19.86% had minimal anxiety. In this study, participants with moderate and severe anxiety were considered to have GAD. This classification highlights that nearly half of the participants (48.93%) met the criteria for GAD.

### 3.2 Background Characteristics of the Participants

Table 1 describes the background characteristics of PCOS‐diagnosed students. The majority were overweight (35.1%) or had a normal BMI (45.7%), while 9.6% were obese and underweight. Most participants were unmarried (73.4%), and nearly all had no children (97.2%) and had not conceived (93.3%) or experienced a miscarriage (96.5%). A significant proportion were smokers (63.8%), and 11% had a family history of PCOS.

**Table 1:** Background characteristics of PCOS‐diagnosed students.

Variables	Categories	Count (*n* = 282)	Percentage
Age in years (18–45)	18–25	198	70.2%
	26–35	51	18.1%
	36–45	33	11.7%
Education level	Undergraduate	208	73.8%
	Postgraduate	74	26.2%
BMI category	Normal weight	129	45.7%
	Obesity	27	9.6%
	Overweight	99	35.1%
	Underweight	27	9.6%
Religion	Hindu	19	6.7%
	Islam	263	93.3%
Marital status	Married	75	26.6%
	Unmarried	207	73.4%
Children status	No	274	97.2%
	Yes	8	2.8%
Conception status	No	263	93.3%
	Yes	19	6.7%
Miscarriage status	No	272	96.5%
	Yes	10	3.5%
Smoking habit	Non‐smoker	102	36.2%
	Smoker	180	63.8%
Family history of PCOS	No	251	89.0%
	Yes	31	11.0%

**Figure 3:**
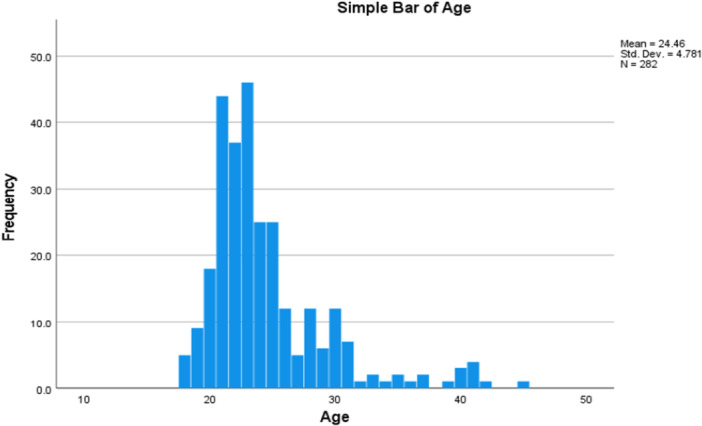
Age distribution of the respondents.

Figure 3 depicts the age distribution of the study population. The mean age of the participants was 24.26 (SD ± 4.781) years, with a minimum age of 18 and a maximum age of 45.

### 3.3 Lifestyle‐Related Characteristics of the Respondents

Table 2 describes the lifestyle and menstrual characteristics of PCOS‐diagnosed females. The menstrual cycle varied among participants, with 36.2% experiencing cycles of less than 3 days and 34.4% lasting more than 7 days. A majority (74.1%) reported irregular periods. Most participants experienced weight gain (68.4%), mood swings (84.8%), loneliness (75.5%), and weakness (70.9%).

**Table 2:** Lifestyle parameters of PCOS‐diagnosed women.

Variables	Category	Count (*n* = 282)	Percentage
Menstrual cycle	3–5 Days	50	17.7%
	5–7 Days	33	11.7%
	Less than 3 days	102	36.2%
	More than 7 Days	97	34.4%
Irregular periods	No	73	25.9%
	Yes	209	74.1%
Weight gain	No	89	31.6%
	Yes	193	68.4%
Mood swings	No	43	15.2%
	Yes	239	84.8%
Loneliness	No	69	24.5%
	Yes	213	75.5%
Lower back pain	No	133	47.2%
	Yes	149	52.8%
Weakness	No	82	29.1%
	Yes	200	70.9%
Treatment	Birth control pills	99	35.1%
	Lifestyle modification	96	34.0%
	Metformin	67	23.8%
	N/A	20	7.1%
Family history of Diabetes/HBP	No	47	16.7%
	Yes	235	83.3%
Physical exercise	No	201	71.3%
	Yes	81	28.7%
Physical exercise weekly	3 days or more	77	27.3%
	less than 3 days	81	28.7%
	Not at all	124	44.0%
Junk foods consumption	Frequently	155	55.0%
	Occasionally	42	14.9%
	Once in a month	6	2.1%
	Once in a week	79	28.0%
Diet pattern	Mixed	142	50.4%
	Non vegetarian	128	45.4%
	Vegetarian	12	4.3%
Meals on time	No	201	71.3%
	Yes	81	28.7%
Skip meals	No	90	31.9%
	Yes	192	68.1%
Water	8 glasses or more	86	30.5%
	Less than 8 glasses	196	69.5%
Birth control	No	246	87.2%
	Yes	36	12.8%
Oral contraceptive pills	No	143	50.7%
	Yes	139	49.3%

Regarding treatment, birth control pills (35.1%) and lifestyle modifications (34.0%) were the most common, followed by metformin (23.8%). A significant proportion had a family history of diabetes or HBP (83.3%).

Lifestyle habits showed that 71.3% did not exercise physically, and 44% never exercised weekly. Junk food consumption was high, with 55% consuming it frequently. Diet patterns were mainly mixed (50.4%) or non‐vegetarian (45.4%). Most participants (71.3%) did not eat meals on time, and 68.1% skipped meals. Water consumption was low, with 69.5% drinking less than 8 glasses daily.

### 3.4 Risk Factors Associated With Generalized Anxiety Disorder Among PCOS‐Diagnosed University Students in Bangladesh

The results of the logistic regression analysis in Table 3 provide essential insights into the determinants of GAD among university students diagnosed with PCOS. The study highlights several significant associations and factors that do not show a statistically significant relationship with GAD.

**Table 3:** Multivariable logistic regression analysis of factors associated with generalized anxiety disorder (GAD) (*n* = 282).

Variables	Factors	Adjusted OR	95% CI	*p*‐value
BMI category	Normal weight	1	Reference	
	Obesity	1.71	(0.68–4.49)	0.27
	Overweight	0.60	(0.32–1.12)	0.11
	Underweight	0.37	(0.12–1.01)	0.06
Marital status	Married	1	Reference	
	Unmarried	2.53	(1.35–4.86)	0.004
Children	No	1	Reference	
	Yes	0.11	(0.01–0.76)	0.03
Mood swings	No	1	Reference	
	Yes	4.18	(1.78–10.6)	0.002
Feel weakness	No	1	Reference	
	Yes	0.56	(0.29–1.06)	0.08
Family history of diabetes/HBP	No	1	Reference	
	Yes	0.388	(0.17–0.85)	0.02
Skip meal	No	1	Reference	
	Yes	2.13	(1.18–3.90)	0.01
Birth control	No	1	Reference	
	Yes	2.84	(1.23–6.98)	0.02
Oral contraceptive pills	No	1	Reference	
	Yes	1.99	(1.14–3.48)	0.02
Age		1.13	(1.06–1.22)	< 0.001

*Note:* Two‐sided tests were used; Level of significance at: *p* < 0.05.

Abbreviations: CI, confidence interval; OR, odds ratio.

Starting with the body mass index (BMI) categories, the analysis reveals that obesity has an adjusted odds ratio (AOR) of 1.71 (95% CI: 0.68–4.49, *p* = 0.27), which is not statistically significant. Being underweight has an AOR of 0.37 (95% CI: 0.12–1.01, *p* = 0.06), indicating a trend toward a reduced likelihood of GAD, though this is not statistically significant. The overweight category, with an AOR of 0.60 (95% CI: 0.32–1.12, *p* = 0.11), also shows no significant association with GAD.

Marital status is a significant determinant of GAD, with unmarried students being more than two and a half times as likely to experience GAD compared to married students (AOR = 2.53, 95% CI: 1.35–4.86, *p* = 0.004). This finding suggests that unmarried students face a higher risk of developing GAD. Additionally, having children is associated with a significantly lower likelihood of experiencing GAD. Students with children are approximately 9 times less likely to develop GAD compared to those who do not have children (AOR = 0.11, 95% CI: 0.01–0.76, *p* = 0.03).

Mood swings are strongly associated with GAD, with students who experience mood swings being more than four times as likely to develop GAD compared to those who do not (AOR = 4.18, 95% CI: 1.78–10.6, *p* = 0.002). This indicates that mood swings are a significant risk factor for GAD in this population. On the other hand, feeling weakness is not significantly associated with GAD (AOR = 0.56, 95% CI: 0.29–1.06, *p* = 0.08), suggesting that weakness may not have a notable impact on the likelihood of developing GAD, though there is a slight trend toward a protective effect.

Family History of Diabetes or HBP is a significant protective factor, with students having a family history of diabetes or HBP being about 2.5 times less likely to experience GAD compared to those without a history of these diseases (AOR = 0.388, 95% CI: 0.17–0.85, *p* = 0.02). This suggests that a Family history of diabetes or HBP may reduce the likelihood of GAD in this population. Skipping meals is significantly associated with a higher likelihood of GAD. Students who skip meals are more than twice as likely to develop GAD than those who do not (AOR = 2.13, 95% CI: 1.18–3.90, *p* = 0.01). This finding underscores the importance of regular eating patterns in managing mental health.

The use of birth control has an AOR of 2.84 (95% CI: 1.23–6.98, *p* = 0.02) and was found to be a statistically significant risk factor for GAD among PCOS‐diagnosed participants, with users being nearly three times more likely to experience GAD compared to non‐users. This indicates a significant association between birth control use and increased risk of developing GAD in this population. Similarly, the use of OCPs is significantly associated with a higher likelihood of GAD, with students using OCPs being almost twice as likely to experience GAD compared to those who do not (AOR = 1.99, 95% CI: 1.14–3.48, *p* = 0.02).

Finally, age is a significant determinant, with each additional year increasing the odds of developing GAD by 13% (AOR = 1.13, 95% CI: 1.06–1.22, *p* < 0.001). This finding indicates that older students with PCOS are more likely to experience GAD.

**Figure 4:**
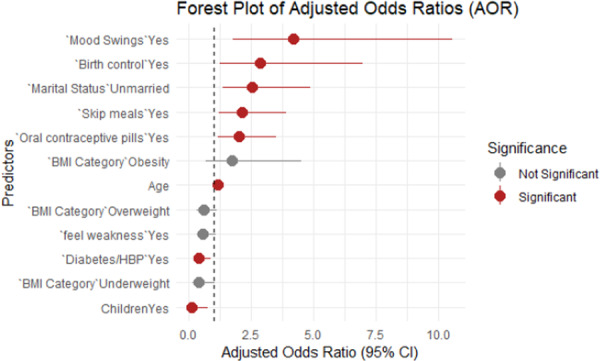
Forest plot of odds ratios.

The finding of the AORs along with their 95% confidence intervals (CIs) is visually presented in a forest plot (Figure 4). The plot highlights the strength and direction of associations for each predictor variable. Factors such as mood swings, birth control, and marital status were significantly associated with an increased risk of GAD. On the other hand, the BMI category, weak feelings did not show a statistically significant protective effect; having children proved to be a protective factor.

## 4 Discussion

The present study focused on risk factors that are potentially associated with GAD among the Bangladeshi female population who were diagnosed with PCOS. In our study, mood swings of the participants were found to be a significant predictor of GAD among students diagnosed with PCOS. This finding aligns with existing literature that mood disturbances are a critical psychological manifestation in women with PCOS, which might result in the possible development of anxiety disorders [11, 25, 33, 34]. PCOS is characterized by a higher prevalence of mental health disorders along with reproductive and metabolic dysfunction [35, 36]. Moreover, females with PCOS frequently face psychological obstacles, including body image dissatisfaction, social stigma, and infertility concerns. The strong association identified in our study is consistent with previous studies that emotional dysregulation substantially increases the risk of GAD in PCOS populations [17, 35], and anxiety and mood disorders serve as both a symptom and a mediator of psychological distress among such patients [36, 37]. It was found in a community‐based longitudinal study in Australian women that infertile women with PCOS were more likely to be anxious, depressed, and to have a higher magnitude of perceived stress [8]. In adults with PCOS, the stress of future life without a child is a psychological factor compromising their health [38]. Similarly, our study outcome depicted that females who had children were less likely to experience GAD. Moreover, research conducted in Lubin City, Poland, revealed that women who had no children had higher severity of depression symptoms compared to women who had children [39]. The majority of infertile women can experience emotional fluctuations, which predispose them to anxiety disorders [34, 40]. Despite the unshared story of infertile women with their family and friends, the prevalence of infertility increases their psychological susceptibility [41]. The attainment of motherhood may contribute to enhanced psychological well‐being, potentially alleviating mood swings, depression symptoms, and anxiety disorders.

In our study, the marital status of the participants emerged as a crucial risk factor that is robustly associated with the occurrence of developing GAD among PCOS‐diagnosed individuals, with unmarried participants being more vulnerable to developing GAD compared to married participants. This finding is in line with previous research demonstrating that unmarried status can greatly influence mental health outcomes, including psychological distress, anxiety, and depression, particularly among women with PCOS [5, 8]. This is because unmarried women with PCOS may face internalized stress from perceived social exclusion, fears of reproductive challenges, which could collectively contribute to anxiety. On the other hand, a study on 409 Bangladeshi PCOS patients displayed opposite findings that the frequency of having GAD was augmented in married individuals compared to unmarried individuals [5]. Nevertheless, a study highlighted that marital status did not affect anxiety levels in women with PCOS [39].

OCPs are the first‐line medical treatment in women with PCOS [42]. The findings of the logistic regression analysis revealed that both the use of birth control and OCPs were recognized as significant risk factors for GAD among students with PCOS. These findings align with existing evidence indicating that contraceptive use may influence mood disturbances, anxiety symptoms, and depression in PCOS‐diagnosed populations [35, 43]. The nationwide prospective cohort study data in Denmark [43]confirmed that hormonal contraception use among females aged 15–34 was associated with depression, and depression was found to be a potential adverse effect of contraceptive use. Females with PCOS are often prone to psychological distress because of hormonal imbalance, metabolic anomalies, and body image concerns.

Furthermore, the use of non‐endogenous hormonal exposure from contraceptive pills may disrupt mood and emotional stability. While literature reported the improvement of health‐related quality of life in PCOS patients taking metformin [44, 45], it is well‐evident that many women stop taking OCPs due to mood changes, especially the onset of depression [46]. However, a large prospective study assessed the impact of 6 months of OCPs use on emotional distress and depression in patients with PCOS and found that the level of depression remained unchanged [47]. Notably, PCOS patients experiencing mood disturbances may be concurrently prescribed antidepressants alongside hormonal ones. Evidence indicates that administration of selective serotonin reuptake inhibitors (SSRIs) and OCPs together may relieve mood and depressive symptoms of PCOS patients, especially those diagnosed with comorbid anxiety or depressive disorders [48].

The contribution of family history of diabetes and HBP as a risk factor for GAD was investigated. Surprisingly, our results revealed that a family history of diabetes and HBP served as a protective factor against GAD among PCOS‐diagnosed students. While a previous study [49] showed that a family history of metabolic diseases, like type 2 diabetes, dyslipidemia, usually increases psychological stress and risk of anxiety, our findings suggest an alternative possibility; individuals familiar with coping mechanisms of chronic disease management within their families may develop greater cognitive resilience and adaptation strategies. Moreover, exposure to family members managing these diseases could foster better health literacy, engagement in healthier lifestyle practices, and emotional preparedness, potentially reducing perceived vulnerability and anxiety among the study participants. It is well‐established from research that higher health literacy among individuals can significantly lower the susceptibility to depression, highlighting its importance in mental health outcomes [50]. On the other hand, another recent study has shown no significant association between a family history of hypertension or diabetes and the presence of anxiety or depression among PCOS patients [25].

This study found a correlation between irregular eating habits, such as meal skipping, and the probable development of GAD. This outcome is consistent with a study in Bangladesh [5], where a higher prevalence of GAD was observed among women who skipped meals regularly. In PCOS populations, skipping meals may heighten emotional vulnerability, which can exacerbate anxiety‐like symptoms [24]. A potential reason for this association is that frequently skipping meals reflects an underlying pattern of chaotic lifestyle and poor self‐regulation, which can function both as a symptom of psychological distress as well as a contributing factor to its development. Additionally, age emerged as a significant determinant, as increased age is tied to developing GAD. Similar findings have been reported in studies by Lin et al., where females with PCOS experienced anxiety‐like behaviors that were associated with age [29]. Besides, a few studies [5, 25] did not observe any association between the age of PCOS females and the occurrence of anxiety or GAD, contrasting our findings.

### 4.1 Limitations

This study was unable to assess the mental health and anxiety concerns over time in cross‐sectional investigations, and causation determination is not possible. Moreover, the application of the Patient Health Questionnaire (PHQ‐9) and the UCLA Loneliness (UCLA‐3) scale for psychometric assessment could yield deeper insights into the mental health status of university‐going participants. The self‐report could influence the study's outcome. Our research was limited to the educated population, and including the masses could provide more accurate data. Furthermore, the enrollment of participants could be enhanced in future studies. Additionally, the absence of a non‐PCOS control group limits direct comparison, though future research with matched controls is encouraged.

## 5 Conclusion

In conclusion, the present study reveals a high prevalence of GAD among university‐going female students with PCOS in Bangladesh. Major risk factors such as mood instability, irregular eating habits, and contraceptive use were significantly associated with increased GAD risk. These findings highlight the need to integrate mental health support into PCOS management, and lifestyle interventions can help in reducing the psychological burden on affected students.

## Author Contributions

**Fardin Al Fahad Shakib:** methodology, data curation, formal analysis, writing – original draft, writing – review and editing. **Ifratul Hasan:** methodology, software, visualization, formal analysis. **Mahmud Hossain:** writing – original draft. **Shaherin Islam Tuba:** investigation. **Nowmy Niaz:** investigation. **Shamim Ahmed:** formal analysis. **Mohammad Borhan Uddin:** conceptualization, methodology, supervision, resources, writing – review and editing, writing – original draft, funding acquisition, project administration. All authors have read and approved the final version of the manuscript.

## Conflicts of Interest

The authors declare no conflicts of interest.

## Transparency Statement

The lead author Mohammad Borhan Uddin affirms that this manuscript is an honest, accurate, and transparent account of the study being reported; that no important aspects of the study have been omitted; and that any discrepancies from the study as planned (and, if relevant, registered) have been explained.

## Data Availability

The datasets supporting the conclusions of this article are included within the article. The authors confirm that the data supporting the findings of this study are available within the article and its supporting materials. Mohammad Borhan Uddin has full access to all the data in this study and takes complete responsibility for the integrity and accuracy of the data and the analysis. The data that support the findings of this study are available from the corresponding author upon reasonable request.
